# Checkpoint Markers and Tumor Microenvironment: What Do We Know?

**DOI:** 10.3390/cancers14153788

**Published:** 2022-08-04

**Authors:** Ramya Ephraim, Sarah Fraser, Kulmira Nurgali, Vasso Apostolopoulos

**Affiliations:** 1 Institute for Health and Sport, Victoria University, Melbourne, VIC 3030, Australia; 2 Institute for Musculoskeletal Science (AIMSS), Melbourne, VIC 3021, Australia

## 1. Introduction

The cancer microenvironment, or tumor microenvironment (TME), describes the non-cancerous cells present in the tumor, such as fibroblasts, immune cells, and cells that comprise the blood vessels and proteins produced by all of the cells present in the tumor that support the growth of the cancer cells [1]. A developing TME is a complicated, dynamic entity [2,3]. Diverse innate and adaptive immune cells that are capable of both pro- and anti-tumorigenic actions infiltrate the site [4]. The dynamic interactions between cancer cells and their microenvironment, which consists of stromal cells (a cellular component) and extracellular matrix components (a non-cellular component), are crucial for promoting the heterogeneity of cancer cells, clonal evolution, and multidrug resistance, which leads to cancer cell progression and metastasis [5]. The nervous system has also been described to interact with cancer cells, a process which is integral to the regulation of tumor growth, angiogenesis, and metastasis [6,7,8]. Following cancer cell transformation and associated cell recruitment, the TME becomes a mechanically complex niche, owing to changes in the extracellular matrix architecture [9]. The development of effective and secure therapeutic approaches to treat cancer can be aided by understanding the underlying cellular and molecular mechanisms driving these connections. This is a novel modality to indirectly disrupt the relationship between cancer cells.

Cancer immunotherapy has progressed at a fast pace in the last years. Immunotherapy for cancer was first described by William B. Coley, who noted tumor shrinkage and disappearance following treatment with a bacterial toxin in the 1890s [10,11]. Since then, it has subsequently been developed into a novel method for treating cancer through boosting the immune system, rather than using chemotherapeutic agents to directly attack the cancer cells [12,13]. This treatment can be broadly classified either as cancer vaccines, adoptive cellular immunotherapy, or therapies using immune checkpoint blockades (checkpoint markers) [14,15]. These markers, which are expressed on cancer cells to be used as an escape mechanism from the immune system, have revolutionized cancer treatment in recent years [16,17,18]. The cells within the TME influence the response to immune checkpoint blockade that covers a range of monoclonal antibody-based therapies aiming to block the interaction of inhibitory receptors (immune checkpoints) expressed on the surface of immune cells, with their ligands [19]. The main targets for these treatments are cytotoxic T-lymphocyte-associated antigen (CTLA-4) and programmed cell death (PD-1) or programmed cell death ligand 1 (PD-L1). The PD-1/PD-L1 axis is at the forefront of interactions between immune, stromal and tumor cells [20,21]. 

## 2. Checkpoint Markers/Inhibitors

**Figure 1:**
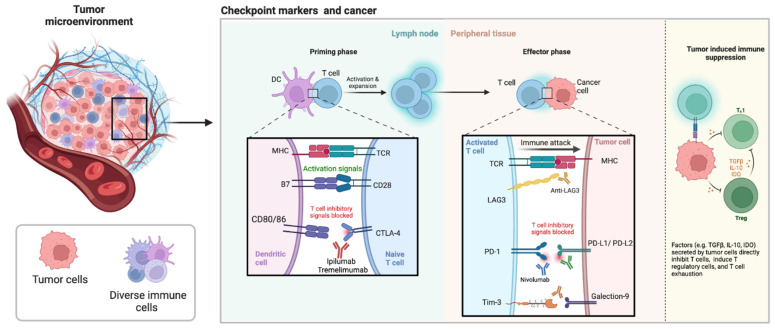
The tumor microenvironment is complex involving diverse immune cells and checkpoint markers. CD, cluster of differentiation; CTLA-4, cytotoxic T lymphocyte associated protein 4; DC, dendritic cells; IDO, indoleamine 2,3-dioxygenase; IL, interleukin; LAG3, lymphocyte activation gene 3; MHC, major histocompatibility complex; PD-1, programmed cell death protein 1; PDL-1, programmed death ligand 1; PDL-2, programmed death ligand 2; TCR, T cell receptor; TGF, tumor necrosis factor; Tim-3, T cell and immunoglobulin mucin domain 3; Treg, regulatory T cells.

Checkpoint markers are expressed on cancer cells as an escape mechanism from the immune system [22]. One of the fundamental roles of the immune system is its ability to differentiate between self- and non-self-antigens and attack the non-self antigens using “checkpoints”. The monoclonal antibody checkpoint inhibitors, CTLA-4 inhibitors (ipilimumab, tremelimumab), PD1/PD-L1 inhibitors (pembrolizumab, nivolumab, durvalumab, atezolizumab), have been routinely used in clinical practice for the treatment of a number of cancers (Figure 1) [23]. As such, checkpoint inhibitors have been used in patients with non-small cell lung cancer, renal cell carcinoma, melanoma, squamous cell carcinoma of the head and neck, urothelial cancer and Hodgkin lymphoma [24]. In contrast to classical chemotherapy, checkpoint inhibitors do not target cancers cells; but rather they enhance activation of immune cells, particularly T cells [16]. In comparison to chemotherapy, tolerance to checkpoint markers is higher with fewer reported side effects. Checkpoint marker inhibitors, improve the outcome of many cancer cases, which may lead to treatment of many patients in future. 

## 3. Role of Immune Checkpoint Inhibitors

The aim of cancer immunotherapy is to promote the activity of CD8+ T cells within the TME to assist the initiation of tumor-specific T cells in lymphoid organs and establish efficient and durable antitumor immunity [25]. To reach optimal effectiveness, many studies have combined treatments that use different targets such as nivolumab (targets PD-1), and ipilimumab (targets CTLA-4) [26]. PD-1 is a checkpoint protein expressed on T cells that act as a type of “off switch” for T cell function. Within last 3 years alone, five new checkpoint inhibitors (all targeting PD-1 or PD-L1), two new cell therapies (targeting CD19), and one new CD3-targeted bispecific antibody (also targeting CD19) have been approved [27] (Figure 1).

**PD-1/PD-L1:** PD-1/CD279 is expressed on activated T cells which binds to PD-L1 or PD-L2 on tumor cells, resulting in inactivation and death of T cells. The absence of PD-1 expression on T cells has shown to significantly delay in tumor growth and increase CD8+ T cells within the TME in mouse models. Nivolumab, is one of PD-1 inhibitors that has been approved by the FDA for use in metastatic melanoma (Figure 1). 

**CTLA-4:** CTLA-4/CD152 is responsible for suppressing CD8+ T-cell activation. Ipilimumab and tremelimumab, are anti-CTLA-4 humanized monoclonal antibodies that have the potential to inhibit CTLA-4 ligand-driven immunosuppression [28]. CTLA-4 and PD-1 are negative regulators of T-cell immune function (Figure 1).

**T cell immunoglobulin and mucin domain-containing protein 3 (TIM-3):** TIM-3 is a negative regulator immune checkpoint. TIM-3 is expressed on IFN-γ producing CD4+ T helper 1 (Th1) and CD8+ T cells (Tc1) as well as on natural killer cells, mast cells, dendritic cells, B cells, macrophages (Figure 1) [29]. TIM-3 is as a target for anticancer immunotherapy since it is expressed on dysfunctional CD8+ T cells as well as on regulatory T cells; two key immune cell populations that constitute immunosuppression within the TME. Blockage of the TIM-3/PD-1 combination is more effective than single blockade in regards to restoring IFN-γ secreting CD8+ T cells [30].

**Lymphocyte-activation gene 3 (LAG-3):** LAG3 is an important immune checkpoint molecule with relevance to several diseases, including cancer [31]. LAG-3 binds to MHC class II in addition to other ligands including galectin-3 and LSECtin. Like PD-1, LAG3 (or CD223) is up-regulated on numerous cell types including, tumor-infiltrating lymphocytes (CD4, CD8) and regulatory T cells. LAG3 is important for optimal T cell regulation and homeostasis [32]. Despite showing that blockade of LAG3 alone was effective at reducing tumor growth this was only weakly effective but blockade of both LAG3 and PD-1 synergistically reduced tumor growth and increased survival of mice [33,34]. Tumor-infiltrating lymphocytes with high LAG3 expression have been noted in solid tumors including, ovarian cancer, melanoma, colorectal cancer, and hematological malignancies including Hodgkin and diffuse large B-cell lymphoma [35]. LAG3 reduces cytokine and granzyme production and proliferation whilst encouraging differentiation into regulatory T cells [31]. 

**Indoleamine 2,3-dioxygenase (IDO):** IDO is an enzyme which degrades tryptophan, an essential amino acid [36] abundant in the lung, colon, and intestine. It catalyzes the oxidative ring cleavage of the pyrrole moiety of not only tryptophan, but also serotonin, melatonin, and other indoleamine derivatives. IDO is expressed by cancer cells but also by endothelial cells, immune cells within the TEM, peripheral blood cells, fibroblasts and in blood [37]. The expression of IDO1 could be activated by the presence of interferon-γ (IFN-γ), lipopolysaccharide, and tumor necrosis factor. Over-expression of IDO1 adjusts the poor prognosis in different types of cancers, such as melanoma, pancreatic, ovarian and colorectal cancers [38].

**Siglecs:** Sialic acid-binding immunoglobulin-like lectins (Siglecs) are transmembrane sialic acid-binding proteins of the immunoglobulin superfamily characterized by the presence of an N-terminal V-set Ig-like domain along with variable numbers of C2 set domains [39]. Siglecs are of interest in cancer since they have been shown to induce immunosuppression, and as such, attractive as targets for anti-cancer effects. Siglecs are primarily expressed by immune cells and thus far 14 siglecs (Siglec1–14) have been identified. Different immune cells express different Siglecs and interact with various ligands on cancer cells. For example, Siglec-7 interacts with salic acid, Siglet-9 with MUC1/MUC16, and Siglec-10 with CD24. Inhibitory Siglecs are Siglec-3, -5, -7, -9, and -10. Siglec-3/CD33 has been studied as a myeloid lymphoma marker in clinical studies. CD33 is associates with myelin-associated glycoprotein (MAG)/Siglec-4 [40]. Siglec-8 is expressed on the surface of human eosinophils, mast cells, and basophils and promotes cytokine-dependent death. Siglec-9 is highly expressed on neutrophils, and induces cell death upon ligation by monoclonal antibodies [41]. 

## 4. Role of TME Cells in Relation to Immune Checkpoint Blockade

Immuno-oncology has come to accept the idea of “hot” and “cold” tumors, where “hot” refers to tumors that contain more inflammatory cells than a specified threshold and “cold” refers to tumors that do not [42]. Immune checkpoint inhibitor medication is ineffective in patients with cold TME, which is characterized by minimal effector T cell infiltration, going from cold to hot TME is essential to enhancing the efficacy of immune checkpoint inhibitor therapy [43]. Tumor-associated macrophages are highly immunosuppressive protecting cancer cells through eliminating the anti-tumor T cells by overexpressing PD-L1, PD-L2, CD80, and Siglec-15 [44]. Immune checkpoint blockade therapies based on the TME have achieved many successes, such as PD-1/PD-L1 and CTLA4 checkpoint inhibitors, which have revolutionized the treatments [45]. Immune checkpoint molecules such as PD-1, IDO-1, LAG-3, and TIM-3 restrict immune function, and tumor cells use them to avoid host immune surveillance [46]. The immune environment supports the invasion and spread of the cancer, and cancer cells have the power to alter the immune system. To engage part in the process of mutual adaptation, these cells also secrete cytokines, immunological checkpoint systems, and tertiary lymphoid structures. Tumor cells interact with the fibroblasts, vascular system, and microbiome [47]. Other products within the TME, including B cells, fibroblast cells, myeloid lineage cells, and the vasculature, also play a part [48]. The other elements of the TME may positively or adversely impact the production of effective antitumor immunity provided the primary objective of successful cancer therapy is to elicit long lasting memory T cell responses [21].

## 5. Conclusions

Determining the relationship between tumor cells and the TME is crucial to developing cancer treatments. Immune checkpoint inhibitors may cause immune-related adverse events, some of which are clinically serious to potentially life-threatening. The establishment of biomarkers that can identify patients who are more likely to benefit from immune checkpoint inhibitor therapy is essential to optimize the use of immunotherapy. The regulation of the TME based on multiple omics results can suggest innovative therapeutic strategies to prevent tumors from succeeding in immune escape and to support antitumoral effects. Bioinformatics approaches will also need to continue to develop, to improve our understanding of a responsive TME. It is well established that the TME affects immune checkpoint inhibitor responses. Predictive and prognostic biomarkers can be determined using cutting-edge technology, which can also be utilized to investigate the immunological background of malignancies.
